# Scan speed control for tapping mode SPM

**DOI:** 10.1186/1556-276X-7-121

**Published:** 2012-02-14

**Authors:** Aleksey V Meshtcheryakov, Vjacheslav V Meshtcheryakov

**Affiliations:** 1 Faculty of Automation and Electronics of the National Nuclear Research University (MEPhI), Moscow, 115409, Russia; 2 Department of Physical and Mechanical Properties Research of Federal State Institution, Technological Institute for Superhard and Novel Carbon Materials, Troitsk, 142190, Russia

**Keywords:** tapping-mode SPM, scan speed, closed-loop control

## Abstract

In order to increase the imaging speed of a scanning probe microscope in tapping mode, we propose to use a dynamic controller on 'parachuting' regions. Furthermore, we propose to use variable scan speed on 'upward step' regions, with the speed determined by the error signal of the closed-loop control. We offer line traces obtained on a calibration grating with 25-nm step height, using both standard scanning and our scanning method, as experimental evidence.

## Background

Tapping mode is considered to be the most precise mode of the scanning probe microscope [SPM] [1-4]. The main disadvantage of this SPM mode is low performance; it takes a long time to obtain the topographic image of the sample surface. The main limiting parameter of increasing imaging speed in tapping mode is the time constant [*τ_c_*] of the cantilever. In contact mode, this limitation is absent. This fact allows the imaging speed to be higher when using, for instance, a high-speed piezoelectric stack actuator [5,6]. However, it's desirable to use tapping mode in many instances since it reduces the lateral forces exerted by the tip on the sample, thereby reducing tip-sample wear [1,4].

The following methods are known to reduce scanning time:

1) The cantilever resonant frequency [*ω*_0_] is increased by reducing cantilever size (and mass) and increasing its stiffness. However, this can be done only by completely changing the probe construction [1].

2) The cantilever quality factor [*Q*] is reduced by means of cantilever external excitation. In this instance, the total signal consists not only of the excitation signal but also of an extra component proportional to the speed of the cantilever deflection. Reducing the cantilever *Q *factor, however, will result in a reduction in the image resolution [3].

3) A dynamic controller (a switching gain proportional-integral [PI] controller) is used on the base of the error signal which increases in a 'parachuting' region [2,4].

The scan speed is assumed constant in each of the above instances. A variable-speed scanning method [7] allows the determination of the scan speed value according to a particular transient response of the PI controller output signal.

In the present paper, we used both the dynamic controller method and variable-speed scanning to obtain the topographic image of the sample surface. In contrast to Zhang et al. [7], the scanning speed was determined by the behavior of the error signal controls (which was the input signal for the PI controller). The PI controller output bandwidth can be determined from the time constant of the loop control. The error signal bandwidth can be determined from the time constant of an AM (or FM) detector of the probe deflection signal. This time constant is an order of magnitude smaller than the time constant of the loop control [1-4]. This allows faster adaptation of the scan speed to a particular sample surface topography.

## Methods

The cantilever oscillation amplitude *A*(*t*), while scanning a step of height Δ *z*, is expressed as [1]

(1) $$A\left(t\right)=A_{\text{sp}}+\Delta z\cdot \left(1-\text{exp}\left(-\omega_{\text{0}}t/2Q\right)\right),$$

where *ω*_0 _is the cantilever resonant frequency, *Q *is the cantilever quality factor, and *A*_sp _is the set point amplitude. Thus, the cantilever transfer function *C*(*s*) takes the form $C\left(s\right)=\frac{1}{\left(1+s\tau_{c}\right)}$, where *τ_c _*is the time constant of the cantilever and is equal to $\tau_{c}=\frac{2Q}{\omega_{\text{0}}}$. The frequency response of the actuator *G*(*s*) and the cantilever deflection signal detector *K*(*s*) has a constant gain equal to DC gain and don't add extra phase lag (it can be assumed that *G*(*s*)·*K*(*s*)· =*G*_0_·*K*_0 _≈ 1) in the bandwidth of interest. Indeed, the pole frequency of the detector transfer function [*ω*_det_] should be at least ten times less than the cantilever resonant frequency $\omega_{\text{det}}=\omega_{\text{0}}\;/)\;10$. The pole frequency of the transfer function *C*(*s*) is equal to ${\tau_{c}}^{-1}=\omega_{\text{0}}\;/)\;2Q\ll \omega_{\text{det}}$ (if Q~100).

Suppose the feedback controller is an integral controller with time constant *τ_i _*whose transfer function *R*(*s*) is $R\left(s\right)=-\frac{1}{s\tau_{i}}$. Then, the frequency-dependent open-loop gain becomes $\left(-\frac{1}{s\tau_{i}}\cdot G_{\text{0}}\cdot K_{\text{0}}\cdot \frac{1}{\left(1+s\tau_{c}\right)}\right)$. Thus, the characteristic polynomial of the loop control's frequency response *D*(*s*) can be written as

(2) $$D\left(s\right)=s^{2}+\frac{1}{\tau_{c}}s+\frac{G_{\text{0}}K_{\text{0}}}{\tau_{c}\tau_{i}}\approx \left(s+\frac{1}{\tau_{c}}\right)\left(s+\frac{G_{\text{0}}K_{\text{0}}}{\tau_{i}}\right).$$

For stability of the loop control, we need to have significantly different frequencies for the real poles of the transfer function:

(3) $$\frac{G_{\text{0}}K_{\text{0}}}{\tau_{i}}\ll \frac{1}{\tau_{c}}.$$

In the case of such characteristic polynomials, the transient response is described by two exponential function, the fast function having time constant *τ_c _*and the slow function, $\frac{\tau_{i}}{G_{\text{0}}K_{\text{0}}}$. As a result, the speed of a closed-loop control system (that is, without loss of surface) is determined by the time constant $\frac{\tau_{i}}{G_{\text{0}}K_{\text{0}}}$. Feedback speed, the speed of the actuator, is limited in tapping mode by the stability condition of the loop control (Equation 3). Thus, the feedback speed is limited by the cantilever time constant *τ_c_*.

Increasing scan speed leads to a loss of surface when a 'downward step' is scanned or a parachuting effect. If an 'upward step' is scanned, it leads to instability of the loop control [1,2].

Let us find the maximum scan speed without loss of surface. The transient response of the loop control to a capacitive displacement sensor output (if the high-frequency pole (frequency ${\tau_{c}}^{-1}$, Equation 2) is ignored) can be written as

(4) $$\frac{\Delta Y\left(s\right)}{\Delta Z\left(s\right)}=\frac{1}{\left(1+s\tau_{i}\;/)\;G_{\text{0}}K_{\text{0}}\right)}.$$

Then, the transient response of the loop control for a downward step of height Δ*z *takes the form

(5) $$\Delta y\left(t\right)=\Delta z\cdot \left(1-e^{-G_{\text{0}}^{}K_{\text{0}}^{}t\;/)\;\tau_{i}}\right).$$

In the latter case the initial vertical actuator speed is

(6) $$\upsilon_{v}=\frac{\Delta y\left(\text{0}\right)}{\Delta t}=\frac{\Delta z\cdot G_{\text{0}}K_{\text{0}}}{\tau_{i}}.$$

Assuming that there is no loss of surface by the probe, the horizontal scan speed *υ_H _*is related to the vertical actuator speed *υ_v _*by

(7) $$\upsilon_{H}=\upsilon_{v}\cdot tg\left(a\;/)\;2\right)=\frac{\Delta z\cdot G_{\text{0}}K_{\text{0}}\cdot tg\left(a\;/)\;2\right)}{\tau_{i}},$$

where *a *is the apex angle of the diamond tip.

From Equation 3, it follows $\frac{\tau_{i}}{G_{\text{0}}K_{\text{0}}}\approx 10\tau_{c}=\frac{20\cdot Q}{\omega_{\text{0}}}$ yielding

(8) $$\upsilon_{H}=\frac{\Delta z\cdot \omega_{\text{0}}\cdot tg\left(a\;/)\;2\right)}{20\cdot Q}$$

An increase in the actuator speed is caused by an increase in the error signal *e*(*t*) = *A*(*t*) - *A*_sp_. For a step of height Δ*z *<*A*_fr_-*A*_sp_, where *A*_fr _is the free-air amplitude (the amplitude of the cantilever oscillation without touching the surface), the error signal is *e*(0) = Δ*z*. That's why the velocity *υ*_H _depends on the step height Δ*z*. For Δ*z *= (*A*_fr_-*A*_sp_), the scan speed becomes

(9) $${\left(\upsilon_{H}\right)}_{\text{lim}}=\frac{\left(A_{\text{fr}}-A_{\text{sp}}\right)\cdot \omega_{\text{0}}\cdot tg\left(a\;/)\;2\right)}{20\cdot Q}.$$

For higher steps, the initial probe speed doesn't increase as the error signal is saturated at *e*_max _= *A*_fr_-*A*_sp_. For scan speed *υ_H _*> (*υ_H_*)_lim_, the tip doesn't touch the surface and loses sample surface.

For example, let us find the scan speed limit for the SPM NanoScan-3D [8] where the probe is a piezoceramic cantilever with a diamond tip. This device allows you to scan the surface topography and to produce indentation and sclerometry simultaneously. If the set point amplitude is *A*_sp _= 0.8·*A*_fr _(where the cantilever free-air amplitude is *A*_fr _= 100 nm), the cantilever resonance frequency is *f*_0 _= 11.5 kHz, the quality factor is 100, and the apex angle of a diamond tip is 120° [8], then the scan speed limit is approximately (*υ_H_*)_lim _≈ 12.5 μm/s.

The loop control is a high-pass filter for the error signal which is related to the height step Δ*z *by $e\left(t\right)=\Delta z\cdot K_{\text{0}}\cdot e^{-\frac{tG_{\text{0}}^{}K_{\text{0}}}{\tau_{i}}}$. In the case of parachuting, the loop control is opened by the loss of sample surface by the probe. The error signal is saturated at *e*_max _= (*A*_fr_-*A*_sp_) ≈ 0.2 *A*_fr_. To avoid, or at least reduce, the parachuting region, the dynamic controller should increase the error signal *e*_max _[2] or reduce the integral controller time constant *τ_i_*.

According to the algorithm implemented on FPGA, if the error signal is more than a threshold *e*_th_, the integrator time constant is reduced according to

(10) $$\tau_{i}\left(t\right)=\tau_{i}-g\cdot \left(e\left(t\right)-e_{\text{th}}\right),$$

where *g *is the 'gain' of the dynamic controller.

As the tip scans over an upward step, the probe oscillation amplitude is reduced. It can be reduced to zero for the height step Δ*z *>*A*_sp _and scan speed *υ_H _*> (*υ_H_*)_lim _(Equation 9). A higher scanning speed can damage both the sample and the tip. A decrease of the time constant *τ_i _*can cause instability of the closed-loop. According to the found algorithm, the scanning speed is reduced for the threshold of the amplitude *A*_low _<*A*_sp_. Scanning at the lower speed is continued as long as the error signal is reduced and the oscillation amplitude is restored.

## Results and discussion

**Figure 1:**
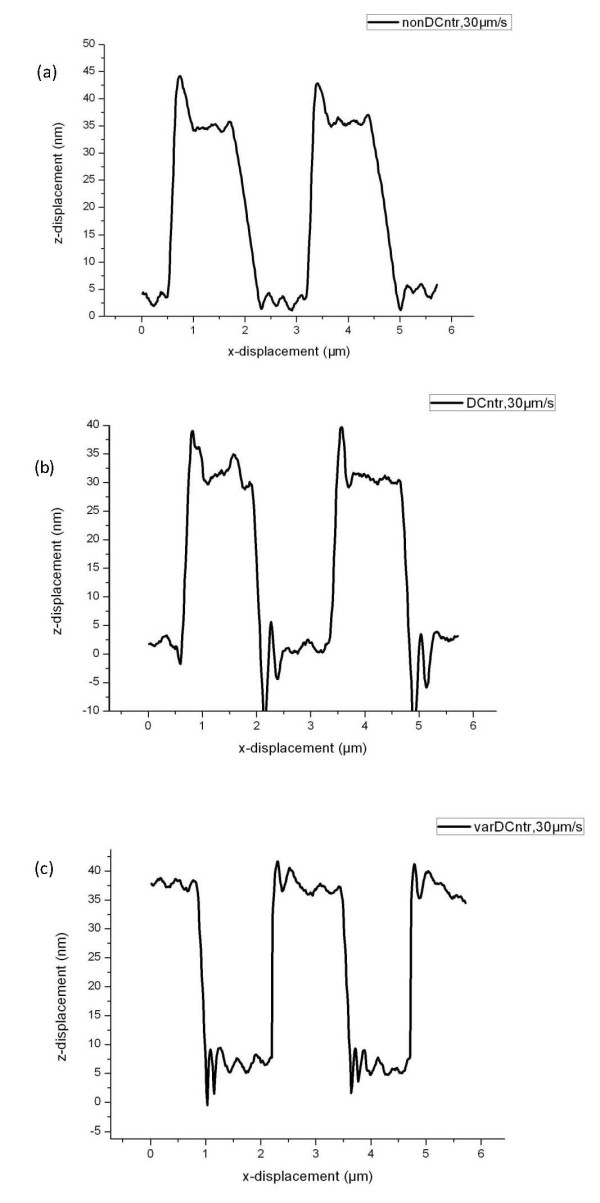
**The line traces of the calibration grating with the step height equal to 25 nm**. At a constant speed of 30 μm/s (a), with a dynamic control (b), and with a dynamic control and at a variable speed (c).

A calibration grating with a step height of 25 nm was used as the sample. A line trace with constant scan speed of 30 μm/s is shown in Figure 1a. A typical scan has a parachuting over a downward step and a peak over an upward step.

The time constant of the implemented dynamic controller is four times decreased in the parachuting region. Figure 1b shows a scan line trace using the algorithm of the dynamic controller. There is practically no parachuting, as shown in the figure. However, the peak over the upward step stayed. In addition, there formed another peak due to a significant increase in the error signal of the loop control after the probe reached the bottom after a downward step. It was decided to reduce the scanning speed in this region.

**Figure 2:**
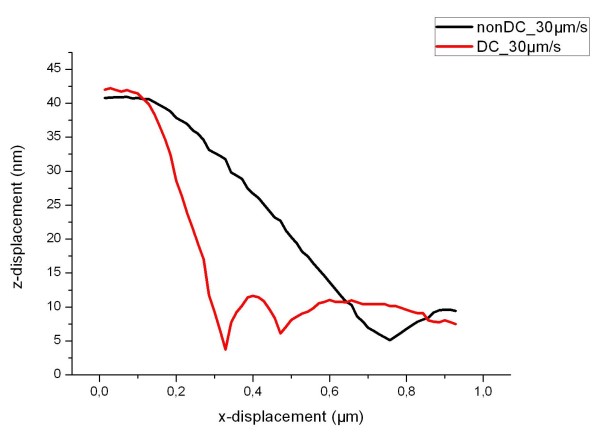
**Comparative line traces for a usual scanning (black) and with a dynamic control (red)**.

Figure 1c shows the line over a downward step trace in the case of a dynamic control and over an upward step for a variable scanning velocity. For a detailed comparison, Figure 2 shows a part of the line traces (parachuting region) in the case of the usual scanning with a constant speed of 30 μm/s and in the case of using dynamic control with variable scanning velocity. For dynamic control, the length of parachuting is reduced by three times.

## Conclusions

The novelty of the presented scanning method consists of using a dynamic controller on a downward step and variable scan speed on an upward step, with scan speed determined by the magnitude of the error signal. As the experimental data on a calibration grating show, assuming equivalent image quality, our method has an advantage of up to three times in imaging speed.

## Competing interests

The authors declare that they have no competing interests.

## Authors' contributions

AVM and VVM contributed equally to this work. All authors read and approved the final manuscript.
